# Perioperative Profiles of Immune Cells in Patients with Postoperative Delirium After Cardiac Surgery with Cardiopulmonary Bypass

**DOI:** 10.3390/biomedicines13122962

**Published:** 2025-12-01

**Authors:** Juan Wu, Zhenzhen Cheng, Xinyi Liao, Ping Yang, Qinjuan Wu, Tingting Wang, Wentong Meng, Zongcheng Tang, Lei Du

**Affiliations:** 1 Department of Anesthesiology, West China Hospital of Sichuan University, Chengdu 610041, China; juanwu266@gmail.com (J.W.); chengzhenzhen@zju.edu.cn (Z.C.); liaoxinyikk@163.com (X.L.); sunflower_yp@outlook.com (P.Y.); 18683378232@163.com (Q.W.); 15982322560@163.com (Z.T.); 2 Department of Anesthesiology, Jiangxi Provincial People’s Hospital, Nanchang 330000, China; 3 Department of Anesthesiology, Chongqing University Three Gorges Hospital, Chongqing 404100, China; 4 Department of Anesthesiology, Chengdu Second People’s Hospital, Chengdu 610041, China; 5 Department of Anesthesiology, Second Affiliated Hospital, Zhejiang University School of Medicine, Hangzhou 310009, China; wtingting@zju.edu.cn; 6 Laboratory of Stem Cell Biology, State Key Laboratory of Biotherapy, West China Hospital of Sichuan University, Chengdu 610041, China; mengwt@scu.edu.cn

**Keywords:** delirium, CD69, antigen presentation, chemotaxis, cardiac surgery

## Abstract

**Background:** Postoperative delirium (POD) is known to involve systemic inflammatory responses, but the characteristics of the immune cell types involved in these responses are unclear. **Methods:** In this prospective study, we compared relative abundances and transcriptomes of circulating immune cells between patients who experienced POD (*n* = 11) or not (*n* = 109) within 7 days after elective cardiac surgery with cardiopulmonary bypass. Blood was sampled before and at 24 h after surgery; features of immune cells were profiled using multi-channel spectral flow cytometry, 10× single-cell RNA sequencing, and measurement of plasma levels of cytokines. **Results:** Patients with POD were older and with higher incidence of congestive heart failure than patients without POD, and these risk factors in turn positively correlated with preoperative proportion of CD40+/HLA-DR+ monocytes and CD69+CD8+ T cells. In addition, preoperative activation of antigen presentation in monocytes and chemotaxis in CD8+ T cells, as well as elevated plasma levels of chemokines CCL3 and CXCL8, were detected in patients with POD. After cardiac surgery, activation of antigen presentation and chemotaxis were also found in patients with POD. **Conclusions:** This study described the perioperative landscape of immune cells in POD and found possible links between preoperative immune dysfunction and risk factors, which may guide future research to explore how the immune system contributes to POD and to design preventive strategies.

## 1. Introduction

Between 4 and 55% of patients who undergo cardiac surgery involving cardiopulmonary bypass experience postoperative delirium (POD) [1]. POD is characterized by fluctuating disorientation in time and space, inattention, and disorganized thinking [2,3]. POD is associated with prolonged hospitalization, longer mechanical ventilation, as well as greater risk of cognitive decline and even mortality [4,5,6].

Numerous studies suggest that POD after cardiac surgery involving cardiopulmonary bypass is driven, at least in part, by systemic immune responses triggered by the combination of surgical trauma, activation of blood components in the extracorporeal circuit, ischemia/reperfusion injury, and endotoxin release [7,8]. For example, patients with POD show higher levels of the pro-inflammatory cytokines interleukin (IL)-2, IL-6, “C-X-C motif” chemokine ligand (CXCL)-8, C-reactive protein, and tumor necrosis factor (TNF)-α in the blood, as well as higher ratios of neutrophils to lymphocytes and monocytes to lymphocytes [8,9,10,11,12,13,14,15]. However, the characteristics of each immune cell type in patients who experience POD after cardiac surgery involving cardiopulmonary bypass are unclear.

Many preoperative risk factors and inflammatory markers related to the occurrence of POD have been identified. Advanced age, higher European System for Cardiac Operative Risk Evaluation II (EuroSCORE II), and male sex have been independently associated with POD incidence [16,17,18]. Similarly, preoperative elevation of inflammatory markers has been associated with the incidence of POD, such as IL-6, CXCL-8, and neutrophil-to-lymphocyte ratio [19,20,21]. However, preoperative profile of immune cells and their relationships with risk factors of POD are unclear.

Therefore, we hypothesized that patients with POD experience comprehensive immune function disturbance both before and after cardiac surgery involving cardiopulmonary bypass, and that preoperative profiles of immune cells are related to POD risk factors. To test these ideas, we prospectively compared patients who experienced POD or not using single-cell RNA sequencing (sc-RNA seq), multi-channel spectral flow cytometry, and protein array analysis from blood sampled before and after cardiac surgery.

## 2. Materials and Methods

### 2.1. Study Design and Patient Enrollment

The observational study was conducted in accordance with the Declaration of Helsinki and approved by the Medical Ethics Committee of West China Hospital, Sichuan University (2022-262) and registered at ClinicalTrials.gov under accession code NCT05400356. Patients who underwent elective valve surgery and/or coronary artery bypass grafting, both involving cardiopulmonary bypass, between 8:00 h and 12:00 h at our medical center between May 2022 and March 2023 were randomly and prospectively recruited into the study according to pre-established inclusion and exclusion criteria (Table S8). Informed consent was obtained from all subjects involved in the study.

### 2.2. Sample Size Calculation

The minimal sample that we planned to recruit was determined by the fact that approximately 10% of patients at our medical center experience POD after elective cardiac surgery involving cardiopulmonary bypass, and the difference in abundance of CD4^+^ T cells between those who do or do not experience POD is approximately (0.22 ± 0.21) × 10^9^/L. Power calculation using R software (version 4.3.0, https://www.r-project.org/, accessed on 30 May 2023) indicated that at least 108 patients should be enrolled in the study to provide 90% power at a two-sided significance of α = 0.05, which we increased to 120 to compensate for an expected dropout rate of 10%.

### 2.3. Anesthesia and Cardiopulmonary Bypass Procedure

General anesthesia was induced with midazolam, propofol, fentanyl, and muscle relaxants, followed by tracheal intubation. Anesthesia was maintained with propofol (6–8 mg/kg/h), remifentanil (0.2–0.3 μg/kg/min), sevoflurane, and intermittent venous muscle relaxants. Cardiopulmonary bypass involved a roll pump (model S5, Stockert, Freiburg im Breisgau, Germany), a membrane oxygenator (Medtronic, Minneapolis, MN, USA), and tubes that had been primed with 750 mL of succinylated gelatin (B. Braun, Melsungen, Germany) and 50 g mannitol. After heparinization (3 mg/kg), bypass was initiated, the aorta was cross-clamped, and the heart was arrested with cold blood cardioplegia. During bypass, blood flow was maintained between 2.2 and 3.0 L/min/m^2^; hemoglobin, above 7 g/dL; nasopharyngeal temperature, 34 °C; mean arterial pressure, 55–80 mmHg; and venous oxygen saturation, 75–85%. At the end of surgery, patients were weaned off bypass and transferred to the intensive care unit.

### 2.4. Assessment of POD and Outcomes

Patients were independently assessed by two investigators (ZCT, MD, and JW, MD, PhD) twice daily for delirium at 8:00–10:00 h and 18:00–20:00 h using a Chinese translation of the Confusion Assessment Method for the Intensive Care Unit (CAM-ICU) if patients were tracheally intubated, or using the conventional CAM otherwise [2,22]. Before tracheally intubated patients were assessed for delirium, their degree of sedation or agitation was assessed using the Richmond Agitation Sedation Scale (RASS). Patients scoring −4 (deeply sedated) or −5 (unarousable) were considered comatose and were not assessed for delirium. Patients were classified into two groups depending on whether or not they experienced POD within seven days after surgery. Investigators who performed follow-up and delirium assessment were trained to use the CAM-ICU and CAM. The training program included lectures introducing delirium and the assessment scales, as well as simulation with actors. Initial training continued until delirium diagnoses reached 100% agreement between investigators. When two investigators disagreed on the diagnosis of delirium, the third investigator decided.

The following outcomes were assessed for all patients during 30-day follow-up: tracheal intubation time, length of stay in the intensive care unit, and all-cause mortality within 30 days or until discharge.

### 2.5. Multichannel Spectral Flow Cytometry

From 120 patients, venous blood samples (8 mL each) before and at 24 h after cardiac surgery were collected into tubes coated with ethylenediamine tetraacetic acid (EDTA) and, within 4 h, treated with an in-house buffer [8.02 g NH_4_Cl, 0.84 g NaHCO_3_ and 0.37 g EDTA disodium salt dihydrate per 1 L of phosphate-buffered saline (PBS); all reagents from Sigma-Aldrich (Saint Louis, MO, USA)] as described [23]. Aliquots of the resulting leukocyte suspension (100 μL) were labeled in separate tubes with antibodies recognizing different markers on the surface of immune cells (Table S9) for 30 min at room temperature in the dark. As a “blank” control, one aliquot was not incubated with any antibody. The aliquots were centrifuged at 300× *g* at 4 °C for 5 min, the supernatant was discarded, the pellet was resuspended in 300 μL PBS, and the suspension was analyzed using the FACSLyric ™ flow cytometer (BD Bioscience, Milpitas, CA, USA). Before loading samples, the manufacturer-installed quality control program was run first, and samples were loaded and analyzed after passing the manufacturer’s quality control. Samples containing single antibodies were analyzed in parallel for the purpose of compensation regulation. Data were analyzed using FlowJo software (Version 10.8.1, Treestar, Woodburn, OR, USA), which was gated to exclude debris, dead cells, and doublets while retaining single live cells. The technician performing flow cytometry was blinded to sample identity.

### 2.6. Identification and Enrichment of Differentially Expressed Genes in 10× Sc-RNA Seq

After matching age, sex, and surgery type between patients who experienced POD or not, we selected two patients who experienced POD and two patients who did not; two aliquots of venous blood (5 mL) from each patient were collected into EDTA-coated tubes, and the resulting eight samples were transferred to the laboratory on ice. Within 8 h after blood collection, peripheral blood mononuclear cells were isolated by centrifugation through a Ficoll Hypaque density gradient. Cell suspensions were used only if viability exceeded 80% based on trypan blue staining.

An appropriate volume of cells was loaded onto a 10X Genomics Chromium instrument to capture 8000 single cells, followed by cDNA amplification and library construction, all according to the manufacturer’s instructions for the 10X Genomics Chromium Single-Cell 3′ kit (version 3; 10X Genomics, Pleasanton, CA, USA). Libraries were sequenced through paired-end 150 bp multiplexing on a NovaSeq 6000 system (Illumina, San Diego, CA, USA) at LC-Bio Technology (Hangzhou, China) to a minimum depth of 20,000 reads per cell. Sequences were analyzed using CellRanger 7.0.0 (10X Genomics), and STAR Aligner (https://github.com/alexdobin/STAR, accessed on 22 June 2023) was used to align sequences to a reference genome while taking splicing into account.

CellRanger output was loaded into Seurat 4.1.1 (https://satijalab.org/seurat/, accessed on 22 June 2023), where data dimensionality was reduced, cell types were clustered together, and sequences were analyzed further. Sequences from multiplet cells were removed using DoubletFinder 2.0.3 (https://github.com/chris-mcginnis-ucsf/DoubletFinder, accessed on 22 June 2023), and the remaining sequences were included in the final analysis only if they came from cells expressing at least 500 genes, of which no more than 25% were mitochondrial. The final analysis of samples before surgery included 11,759 cells from patients who did not experience POD and 14,869 cells from those who did. The final analysis of samples at 24 h after surgery included 9627 cells from patients who did not experience POD and 12,407 cells from those who did.

Results of sequence analysis and gene expression were visualized using the LogNormalize routine within the “Normalization” function in Seurat. Principal component analysis was conducted on normalized expression levels, and the top ten components were used to cluster cells and to perform t-distributed stochastic neighbor embedding. We observed obvious batch effects among the samples, which we corrected using Harmony 0.1.0 (https://github.com/immunogenomics/harmony, accessed on 22 June 2023).

In each immune cell type, differentially expressed genes (DEGs) between patients who experienced POD or not were detected using the bimod test in the “FindMarkers” function of Seurat according to three criteria: (1) log_2_(fold difference) ≥ 0.26, (2) *p* < 0.01, and (3) expression in >1% of cells in both patient groups [24].

The potential function of DEGs was explored by examining whether they were enriched in certain Gene Ontology (GO) terms (www.geneontology.org, accessed on 22 June 2023) relative to the genome background based on the hypergeometric test. DEGs were also examined for enrichment in pathways of the Kyoto Encyclopedia of Genes and Genomes (KEGG) (www.genome.jp/kegg, accessed on 22 June 2023). Enrichment was defined as *p* < 0.05. Gene set enrichment analysis (GSEA) based on GO terms and KEGG pathways was explored using the “clusterProfiler” function in R 4.1.0 (www.r-project.org, accessed on 22 June 2023).

### 2.7. Measurement of Cytokine Levels in Plasma by Protein Chip

Cytokine assays were performed in 50 patients who were the latest to be enrolled, of whom 5 experienced POD and 45 did not. Blood samples were centrifuged at 4000 rpm at 4 °C for 15 min, and 100 mL of plasma was transferred onto a Quantibody Human Inflammation Array 3 (catalog no. OAH-INF-3, RayBioTech, Peachtree Corners, GA, USA), which was incubated overnight at 4 °C. This array can detect 40 cytokines in blood in quadruplicate (Table S10). At room temperature, the array was washed five times with wash buffer I and twice with wash buffer II, incubated for 2 h with the biotinylated antibody cocktail, again washed with buffers I and II as above, incubated for 1 h with Cy3-conjugated streptavidin in the dark, washed again with buffers I and II as above, and allowed to dry in the dark. Cy3 signal at 532 nm was measured using an InnoScan 300 system (Innopsys, Carbonne, France) at a spatial resolution of 10 µm. Signals were normalized to cell number.

### 2.8. Statistical Analysis

Data were analyzed using R 4.1.0 and figures were prepared using Origin 2019b (www.originlab.com/2019b, accessed on 18 November 2022). Continuous variables were checked for normal distribution using the Shapiro–Wilk test, then presented as mean ± SD if normally distributed or as median (25th, 75th percentiles) otherwise. Differences between patients with or without POD were assessed for significance using the *t*-test or Mann–Whitney U test. Categorical data were reported as *n* (%), and intergroup differences were assessed using Fisher’s exact test or the chi-squared test. The ability of proportions of immune cell types to predict delirium was assessed in terms of the area under receiver operating characteristic (ROC) curves. Correlations of variables with one another were explored using Pearson correlation analysis. Unless otherwise noted, statistical results associated with *p* < 0.05 were considered significant.

## 3. Results

### 3.1. Clinical Characteristics and Outcomes

We analyzed immune cells in the blood of 120 patients who underwent elective valve surgery and/or coronary artery bypass grafting. In total, 11 (9.2%) experienced POD within 7 days after surgery. Onset of delirium occurred a median of 29.0 h after surgery and lasted a median of 16.0 h (Table S1). Patients who experienced POD were significantly older, scored higher on EuroSCORE II, and were more likely to have congestive heart failure or thrombocytopenia and to be taking positive inotropes than those who did not, and no patients in two groups received anti-inflammatory agents preoperatively (Table 1). The two groups did not differ significantly in the smoking history, BMI, the distribution of surgery types, or in the duration of surgery or cardiopulmonary bypass. Similarly, the rates of 30-day mortality, duration of tracheal intubation, or length of stay in the intensive care unit were comparable between two groups (Table S2).

### 3.2. Perioperative Activation of Chemotaxis of CD8^+^ T Cells and Pro-Inflammatory State in POD

**Figure 1:**
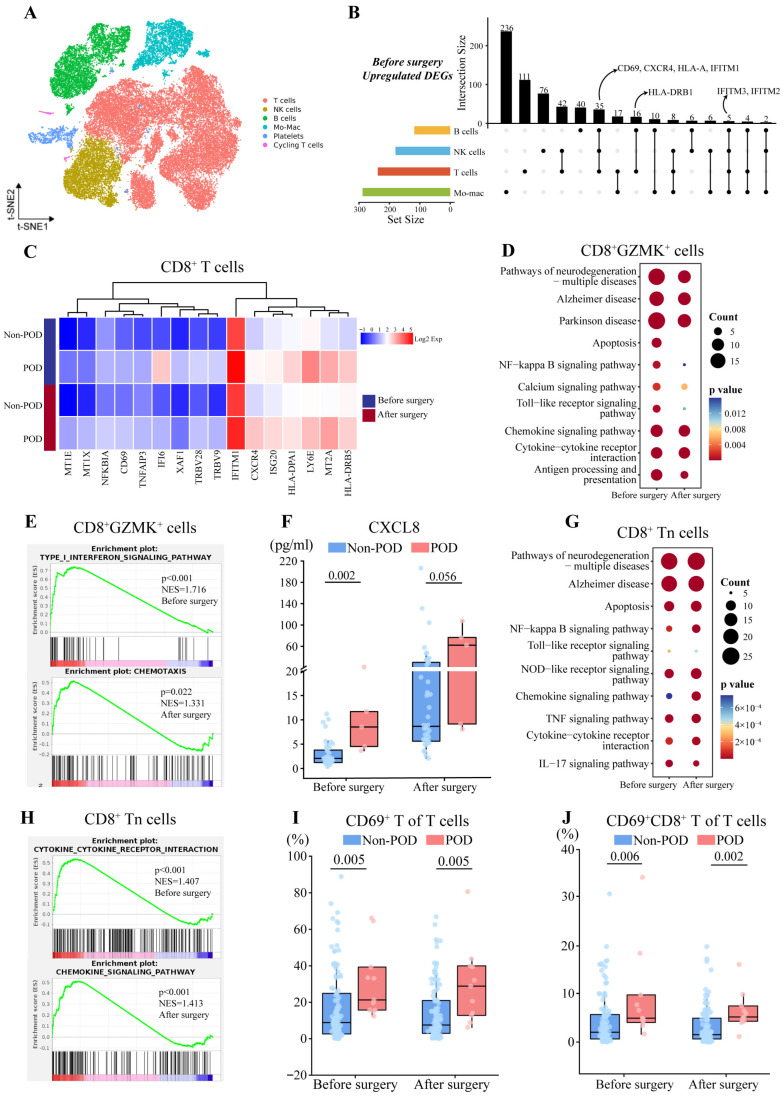
Comparison of transcriptomes and abundance of CD8^+^ T cells between patients who experienced postoperative delirium (POD) or not (Non-POD). (**A**) t-SNE representation of six immune cell subsets identified by single-cell RNA sequencing. Each dot represents a cell, which is colored according to type. Mo-Mac, monocyte-macrophages; NK, natural killer. (**B**) Integrated comparative analysis of perioperative upregulated DEGs in POD group. (**C**) Heatmap of expression of representative DEGs in CD8^+^ T cells between two groups both before and after surgery. Expression levels were log_2_-normalized and represented on a color scale. (**D**) Representative KEGG pathways enriched in DEGs in CD8^+^GZMK^+^ cells before and after surgery. (**E**) Gene set enrichment analysis of DEGs in CD8^+^GZMK^+^ cells before or after surgery, based on Gene Ontology terms. NES, normalized enrichment score. (**F**) Levels of CXCL-8 in plasma based on protein array analysis between two groups both before and after surgery. Differences were assessed for significance using the Mann–Whitney test. (**G**) Representative KEGG pathways enriched in DEGs in CD8^+^ Tn cells before and after surgery. (**H**) Gene set enrichment analysis of DEGs in CD8^+^ Tn cells before or after surgery, based on KEGG pathways. NES, normalized enrichment score. (**I**,**J**) Proportions of (**I**) CD69^+^ T cells and (**J**) CD69^+^ CD8^+^ T cells between two groups both before and after surgery. Differences were assessed for significance using the Mann–Whitney test. DEG, differentially expressed gene; KEGG, Kyoto Encyclopedia of Genes and Genomes; t-SNE, t-distributed stochastic neighbor embedding.

Based on sc-RNA seq, we assigned immune cells in the blood to the following populations based on t-distributed stochastic neighbor embedding (t-SNE) of marker gene expression: T cells, natural killer (NK) cells, B cells, monocyte-macrophages, platelets, and cycling T cells (Figure 1A, Table S3). We identified pre and postoperative differentially expressed genes (DEGs) in T, B, and NK cells as well as monocyte-macrophages between patients who experienced POD or not (Dataset S1), including preoperative upregulation of CD69, CXCR4, and HLA-DRB1 in T and B cells in patients who experienced POD (Figure 1B).

Further, we identified five T cell subpopulations among T cells: CD4^+^ T cells (CD3E^+^CD4^+^), CD8^+^ T cells (CD3E^+^CD8A^+^), γδ T cells (TRDV2^+^), mitochondrial T cells (CD3E^+^MT-CO2^+^), and mucosa-associated invariant T cells (CD8A^+^KLRB1^+^). The expression of CD69, CXCR4, and HLA-DRB1 in CD8^+^ T cells was higher in patients who experienced POD both before and after surgery (Figure 1C), suggesting pre and postoperative activation of CD8^+^ T cells in POD. Within the CD8^+^ T cell subpopulation, we further distinguished three subsets: CD8^+^GNLY^+^ T cells (CD8A^+^GNLY^+^), CD8^+^ naïve T cells (CD8A^+^CCR7^+^), and CD8^+^GZMK^+^ T cells (CD8A^+^GZMK^+^).

CD8^+^GZMK^+^ T cells participate in inflammatory responses and kill target cells by releasing cytotoxic granules [26]. Firstly, we identified 67 preoperative DEGs that also appeared after cardiac surgery (Figure S1A), including upregulated CCL-3 mRNA level, which was similar to the elevated plasma level of CCL3 in patients with POD both pre and postoperatively (Figure S1B). Therefore, we speculated whether patients with POD had preoperatively presented immune dysfunctions related to POD, and then we used pre and postoperative DEGs, respectively, to conduct enrichment analysis. KEGG analysis showed that both preoperative and postoperative DEGs were linked to chemokine- or cytokine-mediated signaling (Figure 1D). GSEA further identified preoperative activation of type I interferon signaling and postoperative chemotaxis in POD patients, with the former suggesting enhanced preoperative cytotoxic function in CD8^+^GZMK^+^ T cells (Figure 1E). Consistent with the activated chemotaxis, we detected elevated plasma levels of CXCL8 before and after surgery in patients with POD (Figure 1F). 

CD8^+^ naïve T cells can be activated to differentiate into cytotoxic T cells [27]. 92 DEGs were identified both before and after surgery in patients who experienced POD, including upregulation of CD69, CXCR4, IFITM1, and NFKBIZ (Figure S1C). Further, both pre and postoperative DEGs were enriched in KEGG pathways related to signaling involving such chemokines or cytokines as IL-17, TNF, and NF-κB (Figure 1G), and GSEA indicated preoperative activation of cytokines–cytokines receptors interactions, and postoperative activation of signaling mediated by chemokines in patients with POD (Figure 1H). Consistent with the enrichment of IL-17 signaling pathway, patients with POD showed pre and postoperative elevation of plasma levels of IL-17A (Figure S1D).

Multichannel flow cytometry was performed in 120 patients both before and after cardiac surgery to validate the results derived from sc-RNA seq. T cells, B cells, monocytes, and NK cells as well as their subtypes were gated according to a gating strategy (Figure S2). We found that before surgery, expression of CD69 on T cells and CD8^+^ T cells, which are markers of early activation [28], were significantly higher among patients who experienced POD than among those who did not (CD69^+^ T cells, 21.40 vs. 8.99%; CD69^+^CD8^+^ T cells, 4.90 vs. 2.00%), and a similar result was obtained at 24 h after surgery (CD69^+^ T cells, 28.90 vs. 7.58%; CD69^+^CD8^+^ T cells, 5.16 vs. 1.51%) (Figure 1I,J, Table S4), which is consistent with the sequencing-based finding of activation of CD8^+^ T cells in POD patients. However, our results did not find any significant correlation between CD8^+^ T cell subsets and plasma cytokine levels (Table S5).

### 3.3. Postoperative Activation of CD4^+^ T Cells and Anti-Inflammatory State in POD

**Figure 2:**
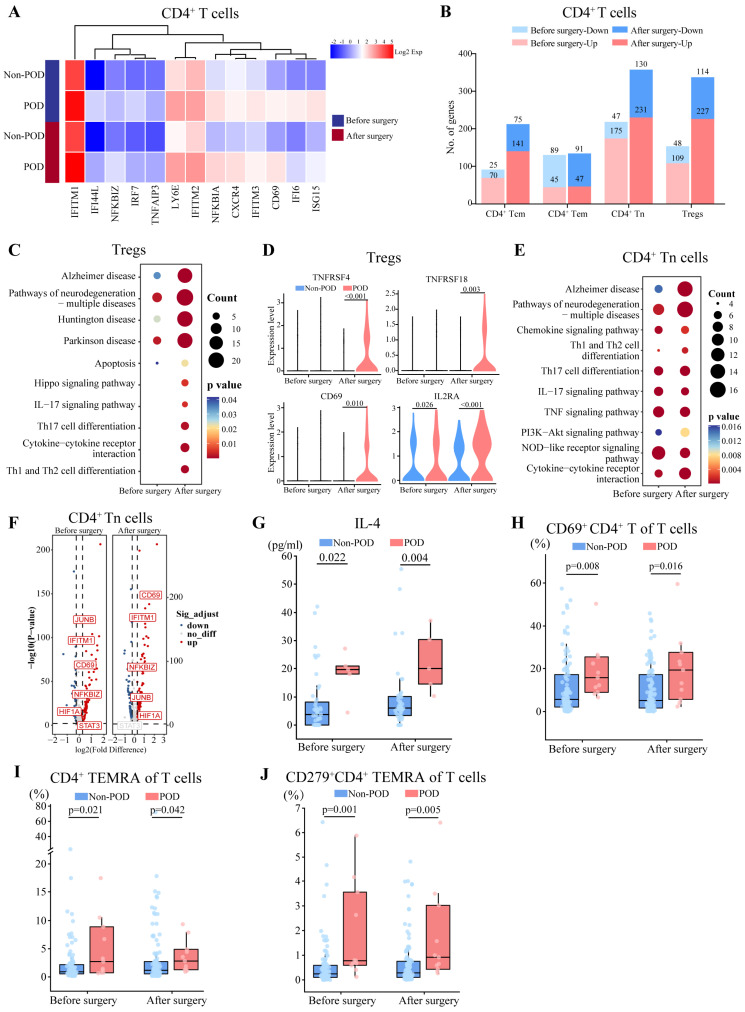
Comparison of transcriptomes and abundance of CD4^+^ T cells between patients who experienced postoperative delirium (POD) or not (Non-POD). (**A**) Heatmap of expression of representative DEGs in CD4^+^ T cells between two groups both before and after surgery. Expression levels were log_2_-normalized and represented on a color scale. (**B**) Numbers of DEGs in CD4^+^ T cell subtypes before and after surgery. Up, upregulation; Down, downregulation. (**C**) Representative KEGG pathways enriched in DEGs in Tregs before and after surgery. (**D**) Relative expression of selected DEGs in Tregs between two groups both before and after surgery. (**E**) Representative KEGG pathways enriched in DEGs in CD4^+^ Tn before and after surgery. (**F**) Volcano plots of selected DEGs in CD4^+^ Tn cells before and after surgery. (**G**) Levels of IL-4 in plasma based on protein array analysis between two groups both before and after surgery. Differences were assessed for significance using the Mann–Whitney test. (**H**–**J**) Proportion of (**H**) CD69^+^CD4^+^ T cells, (**I**) CD4^+^ TEMRA, and (**J**) CD279^+^CD4^+^ TEMRA between two groups both before and after surgery. Differences were assessed for significance using the Mann–Whitney test. IL, interleukin; KEGG, Kyoto Encyclopedia of Genes and Genomes; TEMRA, terminally differentiated effector memory T cells; Tn, naïve T cells; Tregs, regulatory T cells.

CD4^+^ T cells in patients with POD showed upregulation of CD69, IFITM1, and CXCR4 both before and after surgery, suggesting pre and postoperative activation of CD4^+^ T cells (Figure 2A). Among those cells, we were able to distinguish four subsets: regulatory T cells (CD4^+^FOXP3^+^), naïve T cells (CD4^+^CCR7^+^SELL^+^ANXA2^−^), effector memory T cells (CD4^+^CCR7^+^SELL^+^ANXA2^+^), and central memory T cells (CD4^+^GZMA^+^). We focused on regulatory T cells and naïve T cells because they contained the largest numbers of DEGs (Figure 2B, Dataset S2).

Regulatory T cells help maintain immune homeostasis by preventing excessive immune responses [29]. We detected 54 DEGs both before and after surgery in patients with POD (Figure S3A). Further, postoperative DEGs were enriched in KEGG pathways related to differentiation of helper T (Th) cells as well as IL-17-mediated signaling, which was not observed in preoperative DEGs (Figure 2C), and GSEA also indicated postoperative activation of Th1 and Th2 differentiation (Figure S3B). TNFRSF4, TNFRSF18, CD69, and IL2RA were also upregulated in patients with POD after surgery, implying greater differentiation into immunosuppressive regulatory T cells [30] (Figure 2D).

CD4^+^ naïve T cells can differentiate into regulatory and helper T cells after antigen stimulation [31]. We identified 108 DEGs both before and after surgery in patients with POD (Figure S3C). Further, both pre and postoperative DEGs were related to Th cell differentiation, IL-17, and TNF signaling in patients with POD (Figure 2E). Consistent with the enrichment of Th cell differentiation, POD patients showed pre and postoperative upregulation of HIF1A, which could promote Th cell differentiation [32] and elevated plasma levels of IL-4, an anti-inflammatory cytokine secreted by Th2 cells [33] (Figure 2F,G). GSEA further indicated preoperative activation of TNF signaling pathways and postoperative activation of PI3K-Akt signaling pathways, promoting the activation and differentiation of CD4^+^ naïve T cells into Th cells (Figure S3D).

These sequencing-based findings of CD4^+^ T cell activation were supported by flow cytometry, in which POD patients showed significantly higher pre and postoperative relative abundances of CD69^+^ CD4^+^ T cells (Figure 2H). In addition, we observed higher proportions of CD4^+^ terminally differentiated effector memory T cells (TEMRA) before surgery (2.78 vs. 0.94%) and after surgery (2.79 vs. 1.21%), and higher proportions of CD279^+^CD4^+^ TEMRA before surgery (0.77 vs. 0.23%) and after surgery (0.91 vs. 0.28%) (Figure 2I,J, Table S4).

### 3.4. Perioperative Activation of Antigen Presentation and Complement Responses by Monocytes in POD

**Figure 3:**
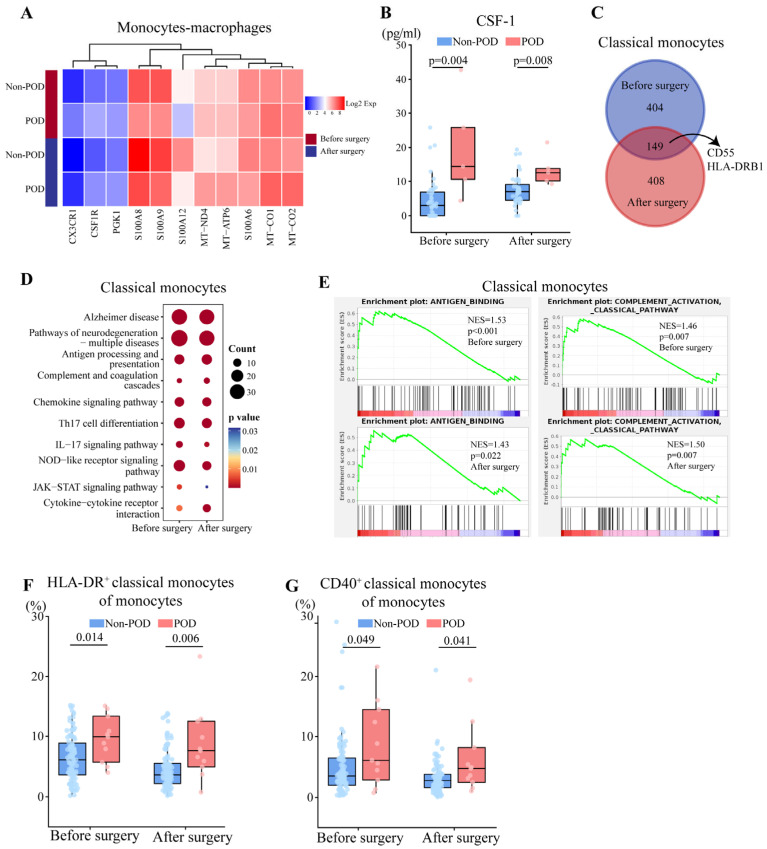
Comparison of transcriptomes and abundance of monocytes between patients who experienced postoperative delirium (POD) or not (Non-POD). (**A**) Heatmap of expression of representative DEGs in monocytes-macrophages between two groups both before and after surgery. Expression levels were log_2_-normalized and represented on a color scale. (**B**) Levels of CSF-1 in plasma based on protein array analysis between two groups both before and after surgery. Differences were assessed for significance using the Mann–Whitney test. (**C**) Integrated comparison of DEGs in classical monocytes between before and 24 h after surgery. (**D**) Representative KEGG pathways enriched in DEGs in classical monocytes before and after surgery. (**E**) Gene set enrichment analysis of DEGs in classical monocytes before or after surgery, based on Gene Ontology terms. NES, normalized enrichment score. (**F**,**G**) Proportion of (**F**) HLA-DR^+^ classical monocytes, and (**G**) CD40^+^ classical monocytes between two groups both before and after surgery. Differences were assessed for significance using the Mann–Whitney test.

POD patients showed both pre and postoperative downregulation of S100 genes and upregulation of CSF1R in monocyte-macrophages (Figure 3A, Dataset S3), and the latter was consistent with the elevated plasma levels of macrophage colony-stimulating factor-1 (CSF-1), the ligand of CSF1R (Figure 3B). Among monocyte-macrophages, we were able to distinguish classical monocytes (CD14^+^ FCGR3A^−^), nonclassical monocytes (CD14^−^ FCGR3A^+^), and macrophages (C1QA^+^).

Classical monocytes detect pathogens, present antigens, and produce cytokines [34]. In patients with POD, we detected 149 DEGs both before and after surgery, including upregulated HLA-DRB1 (essential for antigen presentation) [35] and downregulated CD55 (which prevents complement overactivation) [36] (Figure 3C). Further, we found that both pre and postoperative DEGs were related to antigen processing and presentation, as well as complement and coagulation (Figure 3D), and GSEA also indicated the pre and postoperative activation of antigen binding and the classical complement pathway (Figure 3E).

Consistent with these findings from sc-RNA seq, flow cytometry showed POD patients to have significantly higher pre and postoperative relative abundance of classical monocytes expressing HLA-DR or CD40 (Figure 3F,G, Table S4).

CSF-1, colony-stimulating factor-1; KEGG, Kyoto Encyclopedia of Genes and Genomes.

### 3.5. Perioperative Activation of B and NK Cells in POD

**Figure 4:**
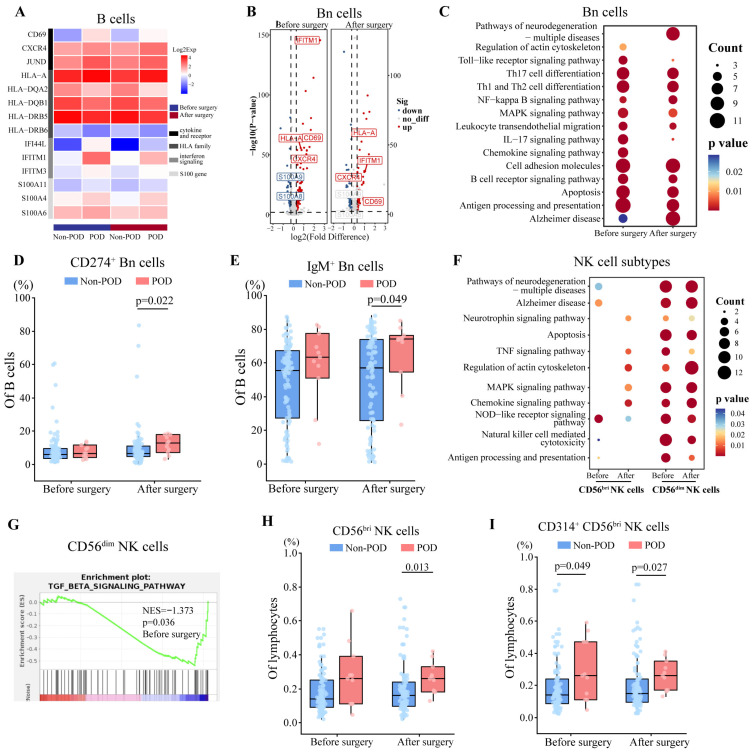
Comparison of transcriptomes and abundance of B cells and NK cells between patients who experienced postoperative delirium (POD) or not (Non-POD). (**A**) Heatmap of expression of representative DEGs in B cells between two groups both before and after surgery. Expression levels were log_2_-normalized and represented on a color scale. (**B**) Volcano plots of DEGs in Bn cells before and after surgery. (**C**) Representative KEGG pathways enriched in DEGs in Bn cells before and after surgery. (**D**,**E**) Proportion of (**D**) CD274^+^ Bn cells, and (**E**) IgM^+^ Bn cells between two groups both before and after surgery. Differences were assessed for significance using the Mann–Whitney test. (**F**) Representative KEGG pathways enriched in DEGs in NK cell subsets before and after surgery. Before, before surgery; After, after surgery. (**G**) Gene set enrichment analysis of DEGs in CD56^dim^ NK cells before surgery, based on KEGG pathways. NES, normalized enrichment score. (**H**,**I**) Proportion of (**H**) CD56^bri^ NK cells, and (**I**) CD314^+^CD56^bri^ NK cells between two groups both before and after surgery. Differences were assessed for significance using the Mann–Whitney test. KEGG, Kyoto Encyclopedia of Genes and Genomes; Bn, naïve B cells; NK, natural killer; bri, bright.

In B cells, 63 DEGs were detected both before and after surgery in patients with POD (Figure S4A), including upregulated CD69, CXCR4, and S100 genes (Figure 4A, Dataset S4), suggesting pre and postoperative activation of B cells. Among B cells, we were able to distinguish three subsets: naïve B cells (CD79A^+^TCL1A^+^), memory B cells (CD79A^+^TNFRSF13B^+^), and plasma cells (CD79A^+^JCHAIN^+^).

In naïve B cells, 64 DEGs were detected both before and after surgery in patients with POD (Figure S4B), including upregulated CD69, CXCR4, HLA-A, and IFITM1 (Figure 4B). Further, we found that both pre and postoperative DEGs were related to antigen presentation and signaling mediated by chemo- and cytokines (Figure 4C). GSEA detected the activation of type I interferon signaling pathways before surgery, and activation of cAMP responses both before and after surgery in POD patients, suggesting the pre and postoperative activation of naïve B cells (Figure S4C). Consistent with these sequencing-based findings of naïve B cell activation, flow cytometry showed POD patients to have had significantly higher postoperative relative abundance of CD274^+^ and IgM^+^ naïve B cells (Figure 4D,E, Table S4). In memory B cells, postoperative DEGs were related to the activation of chemokine receptors and C-C chemokine binding (Figure S4D). In plasma cells, both pre and postoperative DEGs were associated with the activation of type I interferon signaling pathways (Figure S4E).

Based on sc-RNA seq, we identified two subsets of NK cells: CD56^bri^ (NKG7^+^ SELL^+^) and CD56^dim^ (NKG7^+^FCGR3A^+^) NK cells. We identified 9 DEGs in CD56^bri^ NK cells both before and after surgery, and 29 DEGs in CD56^dim^ NK cells (Figure S4F, Dataset S5). Furthermore, postoperative upregulated DEGs were related to chemokine or cytokine signaling in CD56^bri^ NK cells, while both pre and postoperative upregulated DEGs associated with chemokine signaling and NK cell cytotoxicity in CD56^dim^ NK cells (Figure 4F). In CD56^dim^ NK cells, GSEA further indicated preoperative inhibition of TGF-β signaling (Figure 4G), suggesting their preoperative activation [37].

In flow cytometry, we found not only significantly elevated postoperative relative abundance of CD56^bri^ NK cells (Figure 4H), but also the higher expression of CD314 on CD56^bri^ NK cells before and after surgery in POD patients (Figure 4I, Table S4). These suggest pre and postoperative activation of NK cells.

### 3.6. Relationships Between Preoperative Relative Abundances of Immune Cell Types and Risk Factors of POD

**Figure 5:**
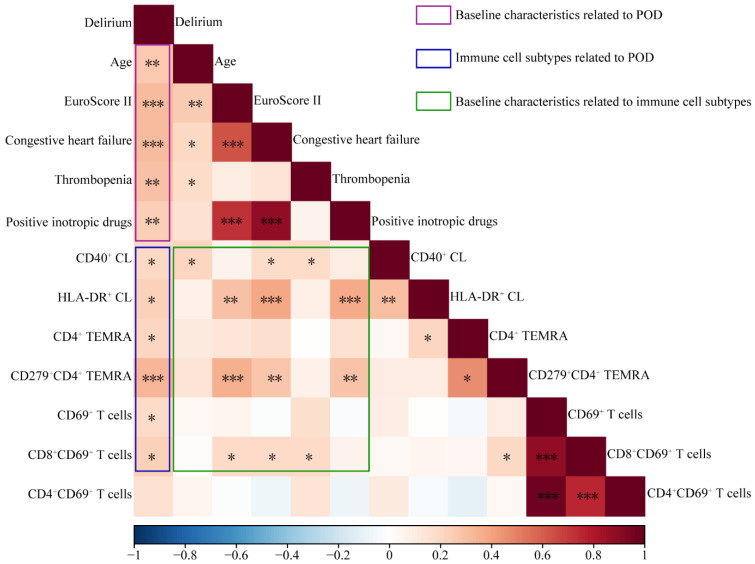
Correlations between preoperative abundances of immune cell populations and preoperative risk factors for postoperative delirium (POD). CL, classical monocytes; TEMRA, terminally differentiated effector memory cells. *, *p* < 0.05; **, *p* < 0.01; ***, *p* < 0.001.

In our cohort, incidence of POD correlated positively with age, EuroSCORE II, preoperative incidence of congestive heart failure, and preoperative use of positive inotropic drugs. Incidence of POD also correlated positively with preoperative relative abundances of CD69^+^ T cells, CD69^+^CD8^+^ T cells, CD4^+^ TEMRA, CD4^+^ CD279^+^ TEMRA, and CD40^+^ classical monocytes (Figure 5, Table S6). These relative abundances showed the following positive correlations with established preoperative risk factors of POD: age correlated with abundance of CD40^+^ classical monocytes; EuroSCORE II, with abundances of CD69^+^CD8^+^ T cells, CD279^+^CD4^+^ TEMRA, and HLA-DR^+^ classical monocytes; congestive heart failure, with abundances of CD69^+^CD8^+^ T cells, CD279^+^CD4^+^ TEMRA, and CD40^+^/HLA-DR^+^ classical monocytes; and use of positive inotropic drugs, with abundances of CD279^+^CD4^+^ TEMRA and HLA-DR^+^ classical monocytes.

## 4. Discussion

As summarized in Table S7, our study provides detailed insights into pre and postoperative alterations in the abundance and behavior of immune cells in patients who experience POD after cardiac surgery with cardiopulmonary bypass. Pre and postoperative alterations include activation of pro-inflammatory processes, of chemotaxis by CD8^+^ T cells, and of antigen presentation by monocytes, while the activation of anti-inflammatory processes was found after surgery. Furthermore, we found that the incidence of POD was associated with elevated preoperative abundance of activated T cells and monocytes, the latter of which was in turn positively correlated with three known preoperative risk factors of POD: advanced age, higher EuroSCORE II, and congestive heart failure. Our results provided a potential explanation for predisposing patients with risk factors to POD, and these findings may guide future research to explore how the immune system contributes to POD and to design strategies to mitigate or prevent it.

Cardiac surgery involving cardiopulmonary bypass triggers systemic inflammatory responses by activating the complement system and release of various cytokines and chemokines as a result of the combination of surgical trauma, activation of blood components in the extracorporeal circuit, ischemia/reperfusion injury, and endotoxin release. These hyperactive immune responses may be related to postoperative complications such as POD and acute lung or kidney injury [7,8]. Our study described the characteristics of systemic immune responses in patients who experience POD after cardiac surgery involving cardiopulmonary bypass, including pro-inflammatory states as well as the activation of chemotaxis and antigen presentation. Previous clinical studies, such as Ko H et al. [12], have linked the elevation of pro-inflammatory cytokines to the occurrence of POD after surgery [8,9,10,11,12,13,14]. The activation of antigen presentation (the first step in lymphocyte activation) as well as widespread lymphocyte activation were observed in our POD patients. The latter phenomenon in turn released numerous cytokines. In addition, anti-inflammatory status was observed after cardiac surgery, as evidenced by activation of Tregs and differentiation of Th2 cells, presumably to counteract the overactivated inflammatory response induced by cardiopulmonary bypass. Furthermore, we observed the activation of chemotaxis by CD8^+^ T cells in patients with POD, and such lymphocytes can cross damaged blood vessels and enter organs under the guidance of chemokine concentration gradients [38,39]. When immune cells migrate into brain parenchyma, neuroinflammation may occur, which is associated with neurocognitive impairment [40]. Therefore, further studies should explore whether the migration of circulating immune cells into the brain parenchyma underlies POD.

The preoperative immune system has exhibited dysregulation among patients who developed POD after cardiac surgery, specifically including the pro-inflammatory states and the activation of monocytes and lymphocytes, which may be associated with the risk factors of POD. Advanced age is an independent risk factor of POD [16], and previous studies have found the association between aging and increased abundance of inflammatory monocytes and upregulation of pro-inflammatory genes [41,42,43], which was consistent with the positive correlation between age and abundance of CD40^+^ classical monocytes in our cohort. CD40 on monocytes is involved in antigen presentation and cytokine secretion [44,45], and moreover, the elevated CSF-1 levels (released by monocytes) among our POD patients also promote inflammatory responses [46]. However, how preoperative antigen presentation is activated in POD patients should be further investigated. One possibility may have to do with the process by which the brain clears antigens, which is that in the central nervous system, potentially threatening antigens in the interstitial fluid are cleared when the fluid drains into the cerebrospinal fluid. This in turn drains into the bloodstream, where the antigens activate monocytes to secrete cytokines and present the antigens to lymphocytes [47].

Congestive heart failure is another independent risk factor of POD in our and previous studies [48,49]. Congestive heart failure could induce systemic inflammatory state, characterized by elevated abundance of immune cells and level of cytokine [50,51]. Furthermore, we found the positive relationship between congestive heart failure and preoperative relative abundances of both CD69^+^CD8^+^ T cells and HLA-DR^+^/CD40^+^ classical monocytes, with the former suggesting T cell activation and the latter involved in antigen presentation and cytokine secretion [44,45,52,53], and these results are consistent with previous studies [54,55,56]. The higher incidence of delirium in patients with heart failure has been investigated, which was associated with the systemic inflammatory state, cerebral hypoperfusion, and impaired cerebrovascular reactivity [57,58]. However, how the dysfunction of monocytes and T cells in congestive heart failure contributes to the occurrence of POD needs further exploration.

Given the following limitations, our results should be interpreted with caution. First, a small sample size, combined with a significant imbalance in group sizes, may raise concerns about potential bias in the results. Second, the limited sample size (*n* = 2 per group) for scRNA-seq may bring potential sampling bias, and thus the results need to be validated in larger cohorts. Third, the examination of only two time points (preoperative and 24h postoperative) may fail to capture delayed neuroinflammatory processes associated with POD pathogenesis, thereby limiting our study to an early postoperative snapshot rather than revealing the complete trajectory of POD development. Fourth, we did not verify or extend most of our bioinformatic analyses through in vitro or in vivo experiments, which should be the focus of future work aimed at clarifying the characteristics of different immune cell types in POD patients.

Despite the above limitations, our study not only described the perioperative alterations in the abundance of immune cells in patients who develop POD after cardiac surgery with cardiopulmonary bypass but also identified the relationship between the abundance of immune cells and risk factors of POD.

## Figures and Tables

**Table 1:** Comparison of preoperative characteristics and surgical parameters between study participants, stratified by whether they experienced postoperative delirium or not.

Characteristic 109	Postoperative Delirium		*p*
	No (*n* = 109)	Yes (*n* = 11)	
Age, yr	56.5 ± 10.2	64.6 ± 9.4	0.018
Male	57 (52.3)	5 (45. 5)	0.908
Body mass index, kg/m^2^	24.0 ± 3.2	24.2 ± 2.9	0.849
Current drinking	27 (24.8)	3 (27.3)	1.000
Current smoking	34 (31.2)	3 (27.3)	1.000
New York Heart Association grade			0.203
I	3 (2.8)	0 (0)	
II	57 (52.3)	4 (36.4)	
III	48 (44.0)	6 (54.6)	
IV	1 (0.9)	1 (9.1)	
EuroSCORE II	1.8 (1.1, 3.0)	3.5 (2.1, 8.0)	0.009
**Comorbidities**			
Congestive heart failure	10 (9.2)	5 (45.5)	0.005
Hypertension	36 (33.0)	3 (27.3)	1.000
Arrhythmia	57 (52.3)	7 (63.6)	0.688
Stroke	4 (3.7)	1 (9.1)	0.387
Diabetes	10 (9.2)	2 (18.2)	0.302
Renal dysfunction ^a^			0.681
Moderate	60 (55.1)	7 (63.6)	
Severe	7 (6.4)	1 (9.1)	
Chronic obstructive pulmonary disease	1 (0.9)	0 (0)	1.000
Peripheral vascular disease	4 (3.7)	2 (18.2)	0.094
Thrombocytopenia ^b^	7 (6.4)	4 (36.4)	0.009
**Current medications**			
Calcium channel blockers	20 (18.3)	3 (27.3)	0.439
Positive inotropic drugs	10 (9.2)	4 (36.4)	0.024
Diuretics	47 (43.1)	6 (54.6)	0.534
Heparin	59 (54.1)	8 (72.7)	0.343
Warfarin	12 (11.0)	3 (27.3)	0.141
**Surgical parameters**			
Type of surgery			
Valve surgery	100 (91.7)	11 (100)	1.000
Coronary artery bypass grafting	6 (5.5)	0 (0)	1.000
Both	5 (3.7)	0 (0)	1.000
Aortic involvement in surgery	26 (23.9)	4 (36.4)	0.464
Duration of cardiopulmonary bypass, min	129 (100, 158)	128 (100, 157)	0.183
Duration of surgery, h	4.3 (3.7, 5.2)	4.8 (4.1, 5.5)	0.310
Transfusion			
Fresh frozen plasma	21 (19.3)	3 (27.3)	0.458
Platelets	27 (24.8)	5 (45.5)	0.160
Red blood cells	20 (18.4)	3 (27.3)	0.439

Values are mean ± SD, median (25% percentile, 75% percentile) or *n* (%), unless otherwise noted. ^a^ Severity was defined as a creatinine clearance rate of 30–59 mL/min (“Moderate”) or <30 mL/min (“Severe”) [25]. ^b^ Defined as platelet count <100 × 10^9^/L.

## Data Availability

The data in this study are available from the corresponding author upon reasonable request. Single-cell RNA sequencing data have been deposited in the Gene Expression Omnibus under accession number GSE252572. Further data will be available from the corresponding author on reasonable request.

## Supplementary Materials

The following supporting information can be downloaded at: https://www.mdpi.com/article/10.3390/biomedicines13122962/s1, Figure S1: Immune profiles of CD8+ T cells in POD patients; Figure S2: Gating strategy in multiple channel flow cytometry; Figure S3: Immune profiles of CD4+ T cells in POD patients; Figure S4: Immune profiles of B cells and NK cells in POD patients; Table S1: Onset and duration of postoperative delirium; Table S2: Comparison of the incidence of outcomes between patients who experienced postoperative delirium (POD) or not (Non-POD); Table S3: Marker expression profiles used to identify immune cell subtypes; Table S4: Comparison of relative abundance of immune cells subtypes and cytokine levels between postoperative delirium (POD) or not (Non-POD); Table S5: Pearson correlation coefficient in correlation analysis between abundance of immune cells subtypes and cytokine level; Table S6: Pearson correlation coefficient and p value in correlation analysis; Table S7: Summary of perioperative immune profiles in patients developed postoperative delirium; Table S8: Criteria for enrolling patients in this study; Table S9: Antibody panel for flow cytometry of immune cell types; Table S10: Organization of the 40 antibodies on the protein microarray. Dataset S1: TBNKMO; Dataset S2: T cells subtypes; Dataset S3: Mo-mac cells subtype; Dataset S4: B cells subtype; Dataset S5: NK cells subtype.
