# Supplementary material for: Perioperative Profiles of Immune Cells in Patients with Postoperative Delirium After Cardiac Surgery with Cardiopulmonary Bypass
Source: Biomedicines. 2025 Dec 1;13(12):2962. doi: 10.3390/biomedicines13122962 (PMC12730709; doi:10.3390/biomedicines13122962)
Supplement: Supplementary file 1 [file biomedicines-13-02962-s001.zip › Tables and Figures.pdf]

Supplementary materials for

Perioperative profiles of immune cells in patients with postoperative delirium after cardiac surgery with cardiopulmonary bypass

Table S1. Onset and duration of postoperative delirium

Patient ID	Onset after surgery, h	Duration, h
POD1	9.0	29.5
POD2	43.0	64.0
POD3	11.0	51.5
POD4	107.0	23.0
POD5	29.0	4.0
POD6	21.0	3.0
POD7	1.5	9.0
POD8	35.0	7.5
POD9	27.0	27.0
POD10	29.0	16.0
POD11	49.0	1.0
**Medians (25% percentile, 75% percentile)**	**29.0 (11.0-43.0)**	**16.0 (4.0-29.5)**

Table S2. Comparison of the incidence of outcomes between patients who experienced postoperative delirium (POD) or not (Non-POD)

Outcome	Non-POD (n = 109)	POD (n = 11)	p
All-cause mortality	1 (0.9)	0 (0)	1.000
Intubation time, h	19.2 (10.6, 21.4)	20 (15.8, 56.0)	0.243
Length of stay in intensive care unit, d	2.9 (1.9, 4.0)	3.9 (2.5, 5.0)	0.130

Values are n (%) or medians (25% percentile, 75% percentile), unless otherwise noted.

Table S3. Marker expression profiles used to identify immune cell subtypes

Cells type	Subtypes	Expression of marker	
		Positive	Negative
T cells	CD4 naïve T cells	CD4 CCR7 SELL	ANXA2
	CD4 effector memory T cells	CD4 CCR7 SELL ANXA2	
	CD4 central memory T cells	CD4 GZMA	
	CD4 regulatory T cells	CD4 FOXP3	
	CD8 naïve T cells	CD8A CCR7	
	CD8 GZMK T cells	CD8A GZMK	
	CD8 GNLY T cells	CD8A GNLY	
	γδ T cells	TRDV2	
	Mucosa-associated invariant T cells	CD8A KLRB1	
	Mitochondria T cells	MT-CO2	
Monocyte-macrophages	Classical monocytes	CD14	FCGR3A
	Non-classical monocytes	FCGR3A	CD14
	Macrophages	C1QA	
Natural killer cells	$CD56^{dim}$ natural killer cells	NKG7 FCGR3A	CD3
	$CD56^{bri}$ natural killer cells	NKG7 SELL	
B cells	Naïve B cells	CD79A TCL1A	
	Memory B cells	CD79A TNFRSF13B	
	Plasma B cells	CD79A JCHAIN	
Platelets		PPBP	
Cycling T cells		MKI67	

Table S4. Comparison of relative abundance of immune cells subtypes and cytokine levels between postoperative delirium (POD) or not (Non-POD)

Result	Before surgery			After surgery		
	POD	Non-POD	p	POD	Non-POD	p
$CD69^{+}$ T cells, %	21.40 (15.65, 36.30)	8.99 (2.67, 24.90)	0.005	28.90 (13.40, 39.70)	7.58 (2.87, 21.10)	0.005
$CD8^{+}$ $CD69^{+}$ T cells, %	4.90 (4.22, 8.80)	2.00 (0.66, 5.73)	0.006	5.16 (4.38, 6.98)	1.51 (0.65, 4.98)	0.002
$CD4^{+}$ $CD69^{+}$ T cells, %	15.70 (10.07, 24.00)	5.56 (1.95, 17.20)	0.008	19.40 (7.86, 25.50)	5.02 (1.49, 17.10)	0.016
$CD4^{+}$ TEMRA, %	2.78 (0.98, 7.82)	0.94 (0.57, 2.22)	0.021	2.79 (1.32, 4.66)	1.21 (0.60, 2.74)	0.042
$CD279^{+}$ $CD4^{+}$ TEMRA, %	0.77 (0.62, 3.09)	0.23 (0.10, 0.58)	0.001	0.91 (0.50, 2.30)	0.28 (0.10, 0.75)	0.005
$HLA\text{-}DR^{+}$ CL, %	9.95 (6.82, 12.15)	6.13 (3.59, 8.83)	0.014	7.59 (5.40, 11.16)	3.58 (2.14, 5.55)	0.006
$CD40^{+}$ CL, %	6.05 (3.69, 13.45)	3.52 (1.99, 6.44)	0.049	4.76 (2.80, 6.84)	2.76 (1.61, 3.83)	0.041
$CD274^{+}$ Bn cells, %	6.49 (4.45, 10.66)	6.04 (3.49, 9.47)	0.424	12.70 (8.02, 17.05)	6.68 (4.65, 10.90)	0.022
IgM^{+} Bn cells, %	63.30 (54.65, 73.35)	55.50 (27.30, 67.20)	0.118	74.10 (63.55, 75.55)	57.00 (25.90, 74.00)	0.049
$CD56^{bri}$ NK cells, %	0.26 (0.11, 0.34)	0.14 (0.09, 0.25)	0.100	0.26 (0.18, 0.31)	0.16 (0.10, 0.24)	0.013
$CD314^{+}$ $CD56^{bri}$ NK cells, %	0.26 (0.13, 0.47)	0.14 (0.09, 0.24)	0.049	0.26 (0.17, 0.33)	0.15 (0.10, 0.24)	0.027
CCL3, pg/mL	1730.57 (1079.61, 1761.46)	587.32 (493.03, 733.25)	0.015	577.26 (519.58, 683.61)	826.55 (806.08, 1021.68)	0.004
CXCL8, pg/mL	1730.57 (1079.61, 1761.46)	587.32 (493.03, 733.25)	0.015	8.64 (5.73, 29.61)	62.42 (9.19, 76.9)	0.056
IL-4, pg/mL	19.72 (18.08, 20.85)	3.70 (0.00, 8.20)	0.024	6.00 (3.63, 10.01)	20.02 (14.54, 30.34)	0.007
IL-17A, pg/mL	14.71 (14.12, 31.44)	2.48 (0.00, 8.42)	< 0.001	9.30 (4.39, 16.02)	34.76 (20.45, 38.3)	0.022
CSF-1, pg/mL	14.37 (10.59, 25.82)	2.96 (0.00, 6.91)	0.004	7.06 (4.52, 8.83)	12.63 (10.24, 13.75)	0.008

Data presented as means ± standard deviations or medians (25% percentile, 75% percentile) unless noted otherwise.

TEMRA, terminally differentiated effector memory cells; CL, classical monocytes; Bn, naïve B cells; NK, natural killer; bri, bright; IL-17A, interleukin-17A; CXCL8, C-X-C motif ligand 8; CCL3, C-C motif ligand 3; CSF-1, colony stimulating factor-1

Table S5. Pearson correlation coefficient in correlation analysis between abundance of immune cells subtypes and cytokine level

**Immune cells subtypes**	**Cytokines (Before surgery)**					**Cytokines (24hours after surgery)**				
	IL-17	MCSF	IL-8	CCL3	IL-4	IL-17	MCSF	IL-8	CCL3	IL-4
$CD4^+$ TEMRA	0.17	0.10	0.22	0.13	0.03	-0.04	0.09	-0.05	0.07	0.10
$CD279^+$ $CD4^+$ TEMRA	0.01	0.04	-0.11	0.04	-0.04	-0.08	-0.10	0.02	0.18	-0.27
$CD274^+$ Bn cells	-0.05	-0.05	-0.10	-0.10	-0.11	0.15	-0.17	0.01	0.19	0.01
IgM^+ Bn cells	-0.02	-0.08	0.04	-0.22	-0.11	-0.12	-0.18	0.02	-0.06	-0.17
$CD40^+$ CL	0.07	-0.17	-0.09	-0.04	-0.18	-0.17	-0.09	-0.10	-0.13	-0.15
$HLA\text{-}DR^+$ CL	0.02	-0.13	-0.06	0.03	-0.16	-0.18	-0.07	-0.22	-0.20	0.05
$CD4^+$ $CD69^+$ T cells	0.00	-0.13	-0.19	-0.11	-0.02	0.00	0.15	0.11	-0.07	-0.02
$CD8^+$ $CD69^+$ T cells	0.00	-0.01	0.18	0.08	-0.09	-0.15	-0.12	-0.02	-0.16	-0.11

Table S6. Pearson correlation coefficient and p value in correlation analysis

	Delirium		Age		EuroScore II		Congestive heart failure		Thrombopenia		Positive inotropic drugs	
	**p**	**r**										
Delirium	0.000	1.00	**p**	**r**								
Age	**0.004**	**0.26**	0.000	1.00	**p**	**r**						
EuroScore II	**0.000**	**0.32**	0.006	0.25	0.000	1.00	**p**	**r**				
Congestive heart failure	**0.000**	**0.32**	0.024	0.21	0.000	0.63	0.000	1.00	**p**	**r**		
Thrombopenia	**0.001**	**0.30**	0.042	0.19	0.239	0.11	0.122	0.14	0.000	1.00	**p**	**r**
Positive inotropic drugs	**0.007**	**0.24**	0.085	0.16	0.000	0.73	0.000	0.88	0.484	0.06	0.000	1.00
CD40$^+$ CL	**0.023**	**0.21**	**0.020**	**0.21**	0.514	0.06	**0.039**	**0.19**	**0.049**	**0.18**	0.236	0.11
HLA-DR$^+$ CL	**0.010**	**0.24**	0.406	0.08	**0.001**	**0.30**	**0.000**	**0.38**	0.363	0.08	**0.000**	**0.38**
CD4$^+$ TEMRA	**0.016**	**0.22**	0.194	0.12	0.142	0.14	0.064	0.17	0.947	0.01	0.067	0.17
CD279$^+$CD4$^+$ TEMRA	**0.000**	**0.34**	0.118	0.14	**0.000**	**0.36**	**0.002**	**0.28**	0.450	0.07	**0.002**	**0.28**
CD69$^+$ T cells	**0.029**	**0.20**	0.681	0.04	0.563	0.05	0.825	-0.02	0.054	0.18	0.798	-0.02
CD8$^+$CD69$^+$ T cells	**0.011**	**0.23**	0.915	0.01	**0.038**	**0.19**	**0.027**	**0.19**	**0.029**	**0.20**	0.472	0.07
CD4$^+$CD69$^+$ T cells	0.067	0.17	0.520	0.06	0.849	-0.02	0.494	-0.06	0.116	0.14	0.428	-0.07

p, p value; r, Pearson correlation coefficient

CL, classical monocytes; TEMRA, terminally differentiated effector memory cells

Table S7. Summary of perioperative immune profiles in patients developed postoperative delirium

Immune dysfunction	Before surgery				24hours after surgery			
	Immune cells subsets	Cytokine	DEGs	Activated or inhibited pathways	Immune cells subsets	Cytokine	DEGs	Activated or inhibited pathways
Activation of chemotaxis AND Pro-Inflammatory state in $CD8^+$ T cells	$CD69^+$T cells, $CD69^+CD8^+$ T cells	CCL3<br>CXCL8<br>IL-17A	CCL3<br>CD69<br>CXCR4<br>IFITM1<br>NFKBIZ	Type I interferon signaling, Cytokines-cytokines receptors interactions	$CD69^+$T cells, $CD69^+CD8^+$ T cells	CCL3<br>CXCL8<br>IL-17A	CCL3<br>CD69<br>CXCR4<br>IFITM1<br>NFKBIZ	Chemotaxis, Chemokine signaling pathway
Anti-inflammatory state in $CD4^+$ T cells	$CD69^+$ $CD4^+$ T cells,<br>$CD4^+$ TEMRA,<br>$CD279^+CD4^+$ TEMRA	IL-4	HIF1A	TNF signaling pathways	$CD69^+$ $CD4^+$ T cells,<br>$CD4^+$ TEMRA,<br>$CD279^+CD4^+$ TEMRA	IL-4	TNFRSF4<br>TNFRSF18<br>CD69<br>IL2RA<br>HIF1A	Th1 and Th2 differentiation, PI3K-Akt signaling pathways
Activation of antigen presentation in monocytes	HLA-DR^+ CL,<br>$CD40^+$ CL	CSF-1	CSF1R<br>HLA-DRB1<br>CD55#	antigen binding, classical complement pathway	HLA-DR^+ CL,<br>$CD40^+$ CL	CSF-1	CSF1R<br>HLA-DRB1<br>CD55#	antigen binding, classical complement pathway
Activation of B and NK cells	/	/	CD69<br>CXCR4<br>HLA-A<br>IFITM1	type I interferon signaling pathway, responses to cAMP	$CD274^+$ Bn cells,<br>IgM^+ Bn cells	/	CD69<br>CXCR4<br>S100 genes<br>HLA-A<br>IFITM1	responses to cAMP, chemokine receptors activity, C-C chemokine binding, type I interferon signaling pathway
	$CD314^+$ $CD56^{bri}$ NK cells	/		TGF-β signaling#	$CD56^{bri}$ NK cells,<br>$CD314^+$ $CD56^{bri}$ NK cells			

#, indicated the DEGs or pathways that were downregulated or inhibited in patients with postoperative delirium.

CL, classical monocytes; TEMRA, terminally differentiated effector memory cells; bri, bright; Th, helper T cells; Bn, naïve B cells; NK, natural killer cells

Table S8. Criteria for enrolling patients in this study

**Inclusion criteria**	(1) At least 18 years old<br>(2) No participation in any other interventional clinical trial within 30 days before surgery or during hospitalization.
**Exclusion criteria**	(1) Pregnancy or lactation<br>(2) History of transplantation of organ(s) or bone marrow<br>(3) Any malignancy<br>(4) Hematological disease, defined as aplastic anemia, thalassemia, acute lymphoid leukemia, acute myeloid leukemia, chronic lymphocytic leukemia, or chronic myeloid leukemia<br>(5) Disease of the immune system, defined as AIDS, Graves' disease, Hashimoto's thyroiditis, systemic lupus erythematosus, nephrotic syndrome, multiple sclerosis, myasthenia gravis, scleroderma, Crohn's disease or ulcerative colitis<br>(6) History of immunosuppressive therapy, defined as monoclonal or polyclonal antibodies, anti-metabolic drugs, alkylating agents, microbial metabolites, or radiation therapy<br>(6) Cognitive dysfunction or mental disorder<br>(7) Inability or unwillingness to provide informed consent

Table S9. Antibody panel for flow cytometry of immune cell types

Fluorophore	Antigen	Antibody description	Source	Catalog no.
		**T cell panel 1**		
BV421	CD279	BV421 Mouse Anti-Human CD279 (PD-1) (MIH4)	BD Pharmingen	564323
BV510	CD25	BV510 Mouse Anti-Human CD25(M-A251)	BD Pharmingen	563352
BV605	CD45	BV605 Mouse Anti-Human CD45(HI30)	BD Pharmingen	564047
FITC	CCR7	FITC Mouse anti-Human CD197 (CCR7) (150503)	BD Pharmingen	561271
PE	CD127	PE Mouse Anti-Human CD127(HIL-7R-M21)	BD Pharmingen	557938
PerCP-Cy5.5	CD3	PerCP-Cy5.5 Mouse Anti-HumanCD3(UCHT1)	BD Pharmingen	560835
PE-Cy7	CD28	PE-Cy7 Mouse Anti-Human CD28(CD28.2)	BD Pharmingen	560684
APC	CD45RA	APC Mouse Anti-Human CD45RA(HI100)	BD Pharmingen	550855
APC-Cy7	CD4	APC-Cy7 Mouse Anti-Human CD4(RPA-T4)	BD Pharmingen	557871
APC-R700	FVS	Fixable Viability Stain 700	BD Pharmingen	564997
		**T cell panel 2**		
BV421	CD279	BV421 Mouse Anti-Human CD279 (PD-1) (MIH4)	BD Pharmingen	564323
BV510	CD8	BV510 Mouse Anti-Human CD8(SK1)	BD Pharmingen	563919
BV605	CD45	BV605 Mouse Anti-Human CD45(HI30)	BD Pharmingen	564047
FITC	CD38	FITC Mouse Anti-Human CD38(HIT2)	BD Pharmingen	555459
PE	CD69	PE Mouse Anti-Human CD69(FN50)	BD Pharmingen	557050
PerCP-Cy5.5	CD3	PerCP-Cy5.5 Mouse Anti-Human CD3(UCHT1)	BD Pharmingen	560835
PE-Cy7	CD28	PE-Cy7 Mouse Anti-Human CD28(CD28.2)	BD Pharmingen	560684
APC	CD45RO	APC Mouse Anti-Human CD45RO(UCHL1)	BD Pharmingen	559865
APC-Cy7	CD4	APC-Cy7 Mouse Anti-Human CD4(RPA-T4)	BD Pharmingen	557871
APC-R700	FVS	Fixable Viability Stain 700	BD Pharmingen	564997
		**B cell panel**		
BV421	CD27	BV421 Mouse Anti-Human CD27(M-T271)	BD Pharmingen	562513
BV510	CD19	BV510 Mouse Anti-Human CD19(SJ25C1)	BD Pharmingen	562947
BV605	CD45	BV605 Mouse Anti-Human CD45(HI30)	BD Pharmingen	564047
FITC	CD38	FITC Mouse Anti-Human CD38(HIT2)	BD Pharmingen	555459
PE	IgG	PE Mouse Anti-Human IgG(G18-145)	BD Pharmingen	555787
PerCP-Cy5.5	IgM	PerCP-Cy5.5 Mouse Anti-HumanIgM(G20-127)	BD Pharmingen	561285
PE-Cy7	CD274	PE-Cy7 Mouse Anti-Human CD274(MIH1)	BD Pharmingen	558017
APC	CD80	APC anti-human CD80	Biolegend	305220
APC-Cy7	IgD	APC-H7 Mouse Anti-Human IgD(IA6-2)	BD Pharmingen	561305

APC-R700	FVS	Fixable Viability Stain 700	BD Pharmingen	564997
		**Monocyte panel**		
BV421	CD284	BV421 Mouse Anti-Human TLR4 (CD284) (TF901)	BD Pharmingen	564401
BV510	CD40	BV510 Mouse Anti-Human CD40(5C3)	BD Pharmingen	563456
BV605	CD45	BV605 Mouse Anti-Human CD45(HI30)	BD Pharmingen	564047
FITC	CD14	FITC Mouse Anti-Human CD14(M5E2)	BD Pharmingen	555397
PE	HLA-DR	PE Mouse Anti-Human HLA-DR(TU36)	BD Pharmingen	555561
PerCP-Cy5.5	CD80	PerCP-Cy5.5 Mouse Anti-Human CD80 (B7-1) (2D10.4)	BD Pharmingen	567437
PE-Cy7	CD274	PE-Cy7 Mouse Anti-Human CD274(MIH1)	BD Pharmingen	558017
APC	CD163	Alexa Fluor 647 Mouse Anti-Human CD163(GHI/61)	BD Pharmingen	562669
APC-Cy7	CD16	APC-H7 Mouse Anti-Human CD16(3G8)	BD Pharmingen	560195
APC-R700	FVS	Fixable Viability Stain 700	BD Pharmingen	564997
		**Natural killer cell panel**		
BV421	CD57	BV421 Mouse Anti-Human CD57(NK-1)	BD Pharmingen	563896
BV510	CD335	BV510 Mouse Anti-Human CD335 (NKp46) (9E2/NKp46)	BD Pharmingen	564064
BV605	CD45	BV605 Mouse Anti-Human CD45(HI30)	BD Pharmingen	564047
FITC	Lin(CD19/CD14/CD123/CD11C/FC)	FITC Mouse Anti-Human CD11c(B-ly6), FITC Mouse anti-Human CD123(7G3), FITC Mouse Anti-Human CD19(HIB19), FITC Mouse Anti-Human CD14(M5E2), Human BD Fc Block (Fc1.3216)	BD Pharmingen	561355,558663,555412,555397,564219
PE	CD127	PE Mouse Anti-Human CD127(HIL-7R-M21)	BD Pharmingen	557938
PerCP-Cy5.5	CD3	PerCP-Cy5.5 Mouse Anti-HumanCD3(UCHT1)	BD Pharmingen	560835
PE-Cy7	CD56	PE-Cy7 Mouse Anti-Human CD56 (NCAM-1) (B159)	BD Pharmingen	557747
APC	CD314	APC Mouse Anti-Human CD314 (NKG2D) (1D11)	BD Pharmingen	558071
APC-Cy7	CD16	APC-H7 Mouse Anti-Human CD16(3G8)	BD Pharmingen	560195
APC-R700	FVS	Fixable Viability Stain 700	BD Pharmingen	564997

Table S10. Organization of the 40 antibodies on the protein microarray

	1[*] 2[*] 3[*] 4[*]	5[*] 6[*] 7[*] 8[*]	9[*] 10[*] 11[*] 12[*]
A	POS1	POS2	CXCL13
B	CCL11	CCL24	GCSF
C	GM-CSF	CCL1	ICAM-1
D	IFN-γ	IL-1α	IL-1β
E	IL-1 RA	IL-2	IL-4
F	IL-5	IL-6	IL-6R
G	IL-7	CXCL8	IL-10
H	IL-11	IL-12 p40	IL-12 p70
I	IL-13	IL-15	IL-16
J	IL-17A	CCL2	CSF-1
K	CXCL9	CCL3	CCL4
L	CCL15	PDGF-BB	CCL5
M	TIMP-1	TIMP-2	TNF-α
N	TNF-β	TNFRSF1A	TNFRSF1B

[*] The antibody against each cytokine was present on the array in four replicate rows.

POS, positive control; CXCL13, C-X-C motif ligand 13; CCL11, C-C motif ligand 11; GCSF, granulocyte colony-stimulating factor; GM-CSF, granulocyte macrophage colony-stimulating factor; ICAM-1, intercellular adhesion molecule-1; IFN-γ, interferon-gamma; IL-1α, interleukin-1 alpha; IL-1 RA, interleukin-1 receptor antagonist; CSF-1, colony stimulating factor-1; PDGF-BB, platelet-derived growth factor BB; TIMP-1, tissue inhibitor of metalloproteinases 1; TNF-α, tumor necrosis factor alpha; TNFRSF1A, tumor necrosis factor receptor superfamily member 1A

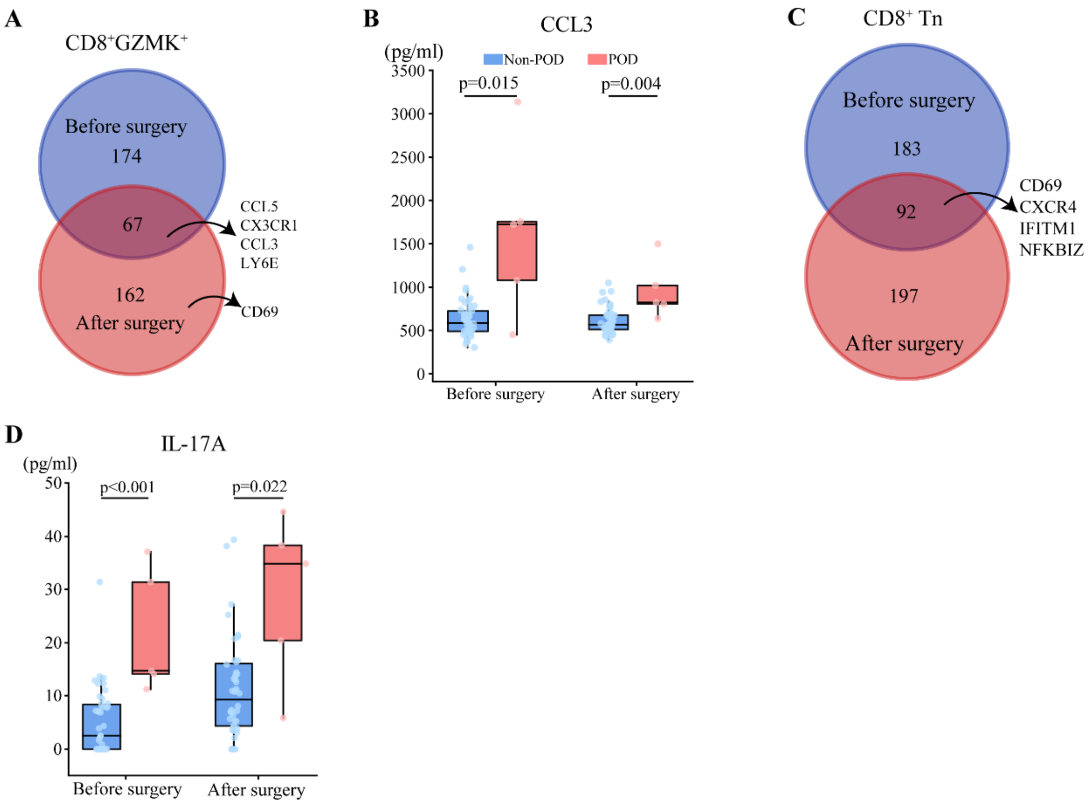

Figure S1. Immune profiles of $CD8^+$ T cells in POD patients.

(A) Integrated comparative analysis of DEGs in $CD8^+$ $GZMK^+$ T cells before and 24 h after surgery.

(B) Levels of CCL-3 in plasma based on protein array analysis.

(C) Integrated comparative analysis of DEGs in $CD8^+$ Tn cells between before and 24 h after surgery.

(D) Levels of IL-17A in plasma based on protein array analysis.

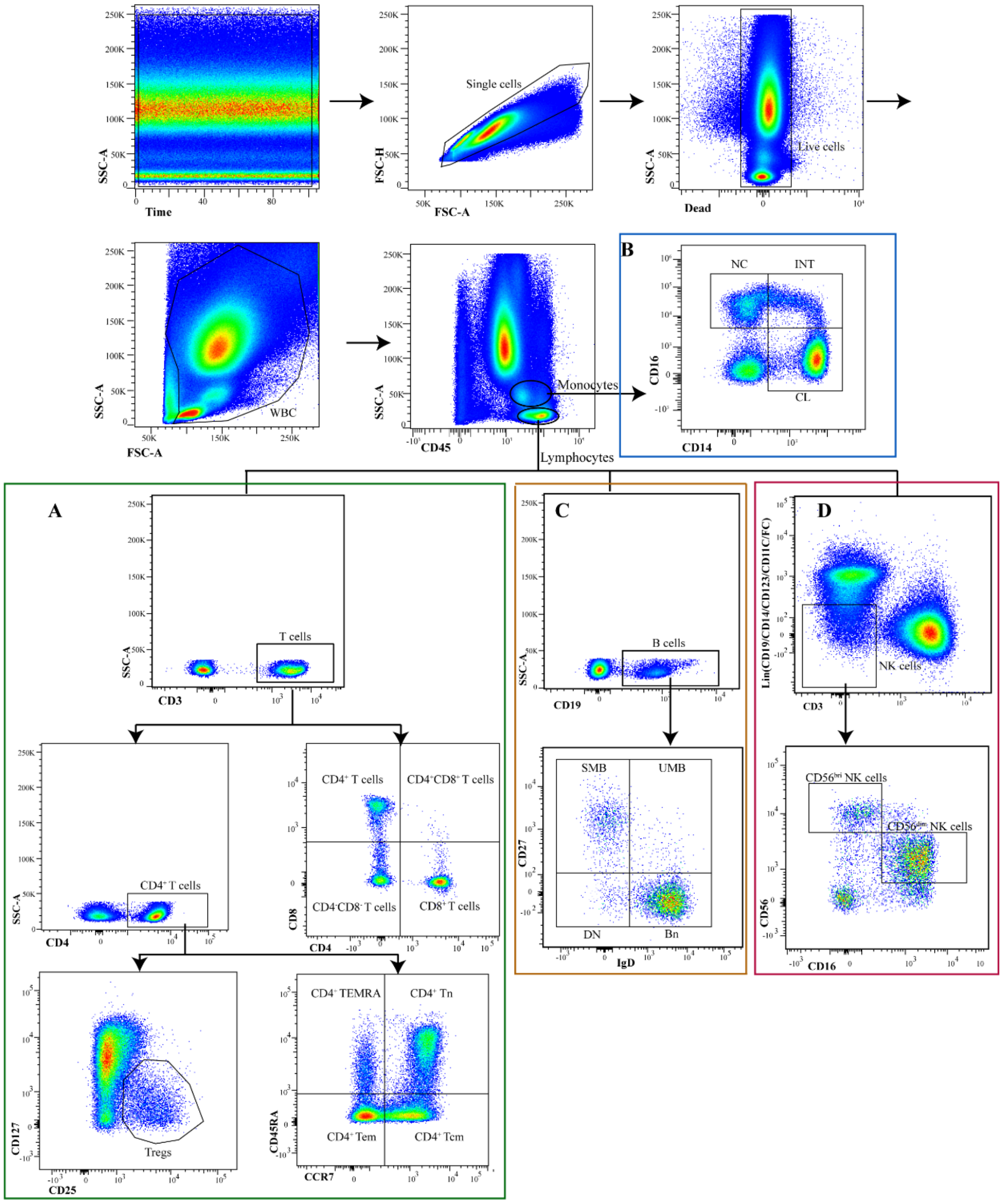

Figure S2. Gating strategy in multiple channel flow cytometry. Gating strategy for (A) T cells, (B) monocytes, (C) B cells, and (D) NK cells.

NK cells, natural killer cells

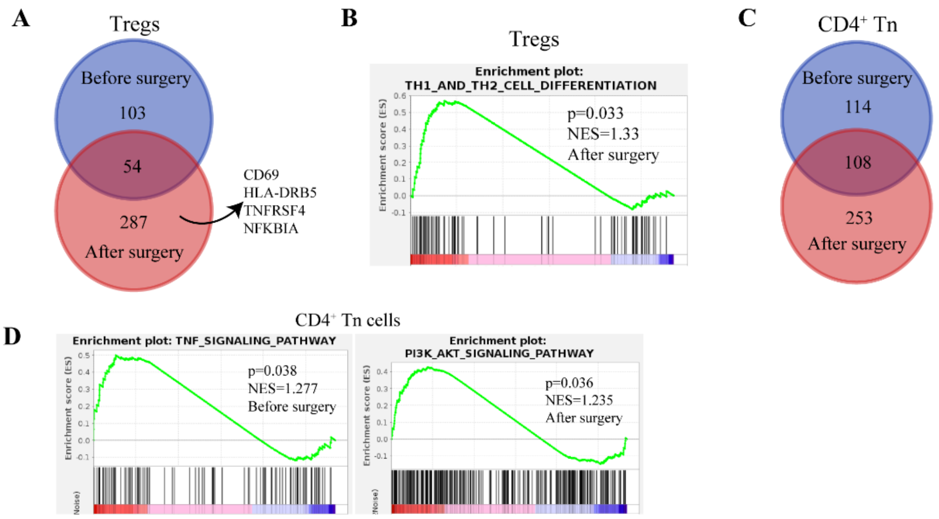

Figure S3. Immune profiles of $CD4^+$ T cells in POD patients.

(A) Integrated comparative analysis of differentially expressed genes (DEGs) in Tregs between before and 24 h after surgery.

(B) GSEA of DEGs in Tregs, based on KEGG pathways. NES, normalized enrichment score.

(C) Integrated comparative analysis of DEGs in $CD4^+$ naïve T cells (Tn) between before and 24 h after surgery.

(D) GSEA of DEGs in $CD4^+$ Tn cells based on KEGG pathways. NES, normalized enrichment score.

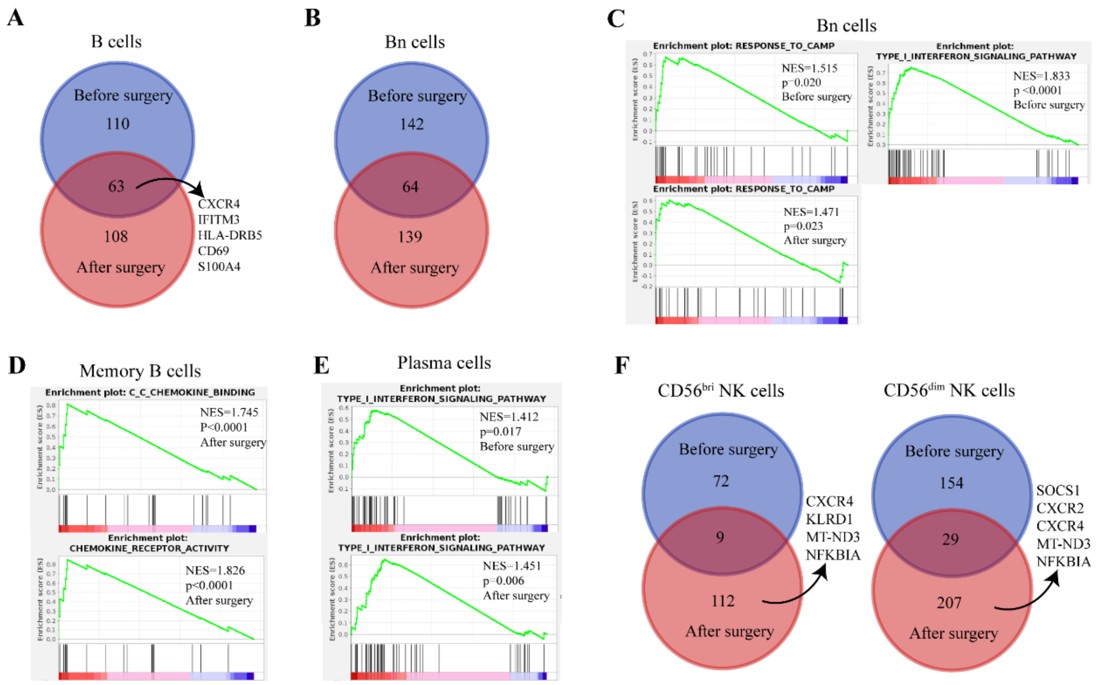

Figure S4. Immune profiles of B cells and NK cells in POD patients.

(A) Integrated comparative analysis of DEGs in B cells between before and 24 h after surgery.

(B) GSEA of DEGs in naïve B (Bn) cells, based on GO terms. NES, normalized enrichment score.

(C) GSEA of DEGs in memory B cells, based on GO terms. NES, normalized enrichment score.

(D) GSEA of DEGs in plasma cells, based on GO terms. NES, normalized enrichment score.

(E) Integrated comparative analysis of upregulated DEGs in NK cell subsets between before and 24 h after surgery.

NK, natural killer cells
